# The Extrastriate Body Area Computes Desired Goal States during Action Planning^1^,^2^,^3^

**DOI:** 10.1523/ENEURO.0020-16.2016

**Published:** 2016-04-05

**Authors:** Marius Zimmermann, Lennart Verhagen, Floris P. de Lange, Ivan Toni

**Affiliations:** 1 Donders Institute for Brain, Cognition and Behaviour, Radboud University Nijmegen, 6525 EN Nijmegen, The Netherlands; 2 Department of Women’s and Children’s Health, Karolinska Institute, 17177 Stockholm, Sweden; 3 Department of Experimental Psychology, University of Oxford, Oxford OX1 3UD, United Kingdom; 4 Max Planck Institute for Psycholinguistics, Radboud University Nijmegen, 6500 HB Nijmegen, The Netherlands

**Keywords:** perception–action, transcranial magnetic stimulation, ventral stream, visuomotor transformations

## Abstract

How do object perception and action interact at a neural level? Here we test the hypothesis that perceptual features, processed by the ventral visuoperceptual stream, are used as priors by the dorsal visuomotor stream to specify goal-directed grasping actions. We present three main findings, which were obtained by combining time-resolved transcranial magnetic stimulation and kinematic tracking of grasp-and-rotate object manipulations, in a group of healthy human participants (*N* = 22). First, the extrastriate body area (EBA), in the ventral stream, provides an initial structure to motor plans, based on current and desired states of a grasped object and of the grasping hand. Second, the contributions of EBA are earlier in time than those of a caudal intraparietal region known to specify the action plan. Third, the contributions of EBA are particularly important when desired and current object configurations differ, and multiple courses of actions are possible. These findings specify the temporal and functional characteristics for a mechanism that integrates perceptual processing with motor planning.

## Significance Statement

It has long been recognized that there must be interactions between the dorsal and ventral cortical streams of visual information processing, but it remains unclear how and when these interactions occur. By studying goal-oriented movements involving the manipulation of an object, we show that the extrastriate body area (EBA), a region in the ventral visuoperceptual stream, provides the dorsal visuomotor stream with a representation of a desired goal state before the dorsal visuomotor stream specifies the motor plan. These findings suggest that perceptual processing, as implemented in the EBA, has functional precedence over visuomotor processing during the selection of goal-oriented movements.

## Introduction

Goal-directed actions are programmed by a dorsal occipitoparietal stream based on visuospatial information. However, flexible manual behavior, especially interactions with objects, often additionally relies on perceptual features. Those features are represented in an anatomically separate occipitotemporal stream geared to process visual material for identification and recognition (Kravitz et al., 2013). In this study, we address a current debate in the literature, namely how and when this perceptual information is used during action planning (Gallivan and Culham, 2015; Lingnau and Downing, 2015). An influential account emphasizes relative differences in processing speeds between dorsal and ventral streams (Milner et al., 2003), and assumes the integration of executive and perceptual processes is time consuming. In this account, the ventral stream could serve as a *post hoc* interpreter of actions, either perceived or planned (Downing and Peelen, 2011; Lingnau and Downing, 2015), or could contribute to action planning, but only when preparation is sufficiently delayed (Rossetti and Pisella, 2002; Cohen et al., 2009). Here, we consider an alternative mechanism, testing whether perceptual features are used as priors by the dorsal visuomotor stream to specify a motor plan (Verhagen et al., 2012). We test this hypothesis by considering an occipitotemporal region in the ventral visual stream, the extrastriate body area (EBA; Downing et al., 2001). This region responds to the perception of body parts, but it is also active during motor planning (Astafiev et al., 2004; Kühn et al., 2011), and it represents a desired postural configuration during actions that require anticipation of future states (Zimmermann et al., 2012). These action-oriented anticipatory abilities are lost in patients with bilateral ventral stream lesions (Dijkerman et al., 2009).

We address these hypotheses using time-resolved neural interference with transcranial magnetic stimulation (TMS) in a rigorous four-level factorial design that controls for the current and intended object state, for the number of possible courses of action, for the time of interference, and for the site of interference. We asked participants to grasp a bar and rotate it to match a target orientation, exploiting the fact that when multiple end-state postures are possible, participants prioritize postural comfort in the final state of the action. Using this motor control strategy involves selecting a comfortable body posture compatible with the final configuration of an object, necessitating the anticipation of that object configuration (Rosenbaum et al., 2001, 2012). A neural implementation of this anticipatory strategy requires sensorimotor transformations that integrate predicted hand–object configurations with biomechanical constraints and the current body posture. We hypothesized these transformations to be causally supported by neural activity in either EBA or in the caudal section of the intraparietal sulcus (IPS), a portion of the dorsal visuomotor stream involved in integrating current body posture with motor plans (Wolpert and Ghahramani, 2000; Zimmermann et al., 2012). To probe the temporal and causal dependences of these ventral and dorsal stream areas, we used single-pulse TMS to disturb processing in EBA and IPS at different time points during the planning of actions with different demands on grip- and end-posture selection.

If EBA provides the dorsal visuomotor stream with a desired goal posture for actions that involve object manipulations and require anticipation of future postural states (van Nuenen et al., 2012; Zimmermann et al., 2012), then TMS to EBA should be particularly disruptive during early stages of motor planning, before the involvement of the dorsal visuomotor stream, especially when the action allows choices between different possibilities. We contrast this hypothesis with other accounts predicting that EBA computations are inconsequential for motor behavior (Downing and Peelen, 2011) or are relevant only during late planning stages of actions (Milner and Goodale, 2008).

## Materials and Methods

### Overview

**Figure 1.**
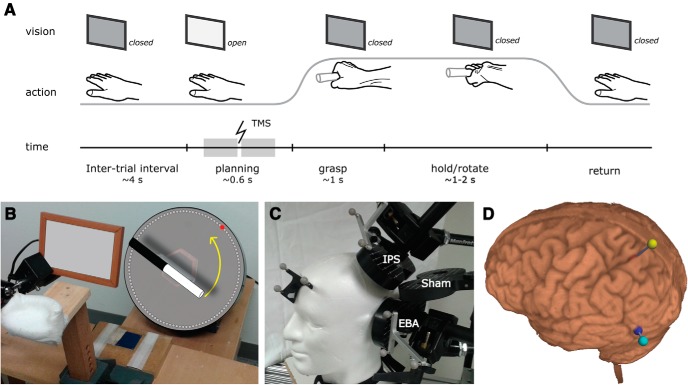
Experimental setup. ***A***, ***B***, Participants grasped the bar around the border between the black and white parts, and rotated it to align the white part with the red LED. On each trial, subjects decided how to grasp the bar: their thumb could be on the white or on the black end of the bar (toward and away grips, respectively). At trial onset, an electronic shutter (***B***, in white, brown frame) allowed the participant to see the bar and the target LED. A single TMS pulse was delivered either early (100–300 ms) or late (300–500 ms) during the planning phase (***A***, gray blocks on time line). When the participant moved his right hand from a home key (***B***, in blue), the electronic shutter became opaque, preventing vision of the hand, of the bar, and of the target LED. ***C***, The participant kept his chin against a chin rest, and his head position relative to the TMS coils was continuously monitored with a video-based frameless stereotaxic system. ***D***, The brain location of the TMS targets (EBA, in blue; IPS, in red) relative to the closest scalp location (in cyan and yellow, respectively). On each trial, TMS was delivered only when the TMS coils were positioned within 5 mm from the desired scalp locations.

The experiment consisted of four experimental sessions (i.e., one intake session and three TMS sessions). During the intake session, participants practiced the experimental task, underwent a short TMS session to determine their motor threshold, were familiarized with stimulation of the EBA and IPS, and participated in a number of MR scans. The three TMS sessions followed the same procedure: after TMS stereotaxic registration and coil placement, participants performed a motor task while being stimulated with single-pulse TMS on a trial-by-trial basis delivered through one of three coils placed on their head (Fig. 1C). Measures of task performance collected on a trial-by-trial basis (see TMS procedures) were used to assess the consequences of TMS on EBA and IPS. The study was approved by the local ethical commission (CMO Arnhem-Nijmegen). For clarity, experimental factors are marked in small caps, and conditions within experimental factors are marked in UPPER CASE.

### Participants

Thirty-one healthy, right-handed participants gave written informed consent to participate in the study and were financially compensated with a payment of 10 euro/h. This study is based on the 24 participants (mean age, 24 ± 3 years; 13 male) that completed the experiment. Seven participants did not complete the whole set of experimental sessions for various reasons (one participant was unable to maintain his/her head in a sufficiently stable position; two participants repeatedly missed their appointments, two participants developed a mild headache, two participants felt otherwise uncomfortable). Two subjects were excluded from the analyses due to responses systematically above the reaction time cutoff (see Motor task). This study is based on the 22 remaining participants (mean age, 24 ± 3 years; 13 male).

### Motor task

The participants followed a single set of instructions across two experimental factors with two conditions each (action-type: HOLD, ROTATE; grip options: SINGLE, MULTIPLE). They were asked to grasp a bar (length, 30 cm; diameter, 2 cm; one end colored black, the other end white) using a power grip with their right hand, around the border between the black and white portions of the bar, and to rotate it in order to align the white portion of the bar with 1 of 256 light-emitting diodes (LEDs) distributed in a circle around a low-friction turning platform supporting the bar (Fig. 1B). Two metal rods of 10 cm length connected the bar with the turning platform. The distance between the rods was 13 cm, allowing participants to grasp the bar in between the rods. On each trial, the bar could be grasped in two ways (i.e. with the thumb on the white or on the black end of the bar). Grips with the thumb placed on the white end of the bar are arbitrarily labeled as “toward” (e.g., with the thumb toward the target LED), and those with the thumb placed on the black end as “away” (e.g., with the thumb away from the target LED).

Each participant executed a total of 1278 trials (426 trials per session), pseudorandomly intermixed across conditions. In each session, trials were performed in nine blocks of maximally 50 trials each. Within the factor action-type, there were ROTATE trials (*n* = 483, the bar was to be rotated by 180°) and HOLD trials (*n* = 483, the bar was to be held around its original orientation). There were also filler trials [the bar was to be rotated by 90° clockwise (*n* = 156) or 90° counterclockwise (*n* = 156)], which were introduced to reduce task predictability but were excluded from subsequent analyses. In order to further avoid the development of stereotyped movement patterns and to trigger a novel action plan on each trial, the target displayed in each trial was extracted from one of four uniform distributions of targets with an average of 0°, ±90°, 180° and a range of ±7° for HOLD, filler, and ROTATE trials, respectively. Put differently, the bar needed to be rotated in all conditions, including the HOLD condition, and to different extents even within trials of the same condition. Slow responses (reaction time >1000 ms) and large errors in bar placement (>36°) were marked as incorrect trials and excluded from the analyses.

**Figure 2.**
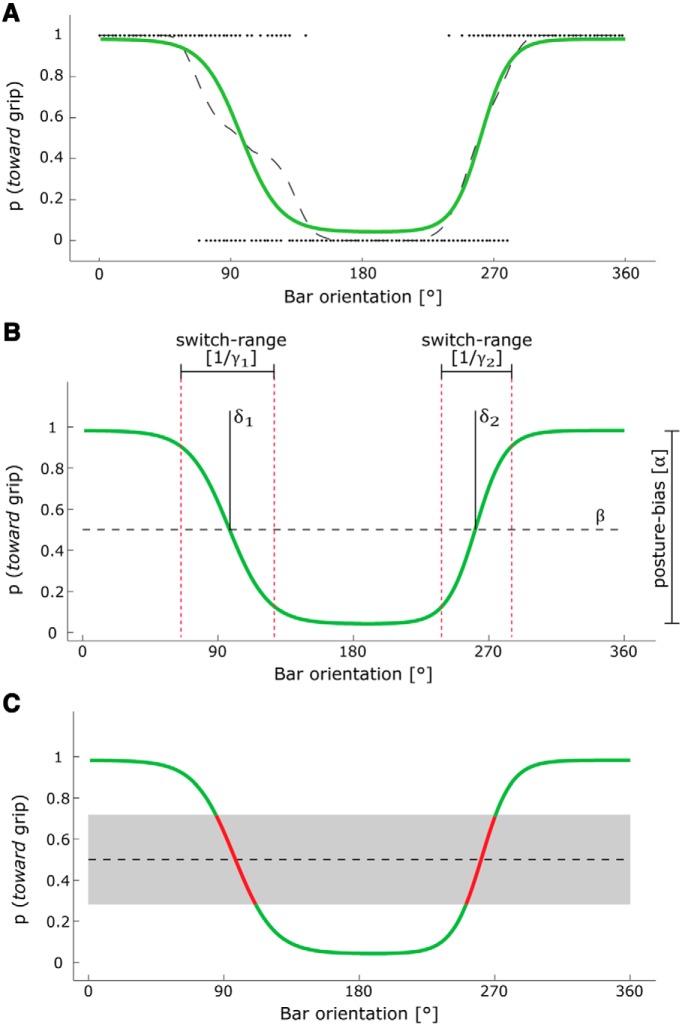
Psychometric analysis of grip choice. A psychometric procedure quantified the probability of selecting one of two possible grips [*p* (toward grip)] as a function of the orientation of the bar (in degrees; 0° corresponds to an upward pointing bar/12 o’clock position, with the number of degrees increasing clockwise). ***A***, ***B***, Single-trial choices (dots) were summarized in a grip choice profile with a moving average (***A***, dashed line) and parameterized with a psychometric function (***A***, ***B***, green line) given by the sum of two logistic curves, resulting in six parameters (***B***): an amplitude parameter (∝) capturing to what extent grip choice is influenced by the expected body posture toward the end of the action (posture bias); an offset parameter (β) capturing biases toward either grip type, irrespective of the bar orientation (systematic bias); two slope parameters (γ_1_, γ_2_) capturing the range of bar orientations over which participants switch their grip preference from one type to the opposite grip type (switch range); and two phase-offset parameters (δ_1,_ δ_2_) capturing the orientations at which participants switch their grip preference from one type to the opposite grip type. ***C***, This panel illustrates how trials with a bar orientation evoking equally mixed grip preferences [*p* (*toward* grip) = ∼0.5] were classified as MULTIPLE-option (red line). The cutoff value for this category of trials (gray interval) was estimated separately for each participant and included 25% of the trials. The remaining trials evoked consistent grips and were classified as SINGLE option.

The motor task included a second experimental factor, grip options, with two conditions (SINGLE, MULTIPLE). This factor accounts for the fact that in this task, given human biomechanical constraints and end-state comfort effects (Rosenbaum et al., 2006), some object orientations allow for both toward and away grips (MULTIPLE-option trials), whereas other object orientations evoke a clear preference for a particular grip (SINGLE-option trials; Fig. 2C). The number of trials contributing to the single- and multiple-option categories was matched between participants by considering 25% of the trials as MULTIPLE option and classifying the remaining 75% as SINGLE option. This ratio was based on the observation that the switch range for grip selection (Fig. 2B) equaled ∼25% of the full orientation range.

The trial timecourse (Fig. 1A) was as follows: at trial onset, the opening of a liquid crystal display (LCD) shutter screen allowed the participant to see the bar and a target LED. As soon as the participant moved his right hand from a touch sensor positioned along his midsagittal plane (Fig. 1B), the screen turned opaque again, preventing vision of the hand, of the bar, and of the target. At the end of each trial, when the participant positioned his right hand again on the touch sensor, the shutter remained opaque, while the bar was silently and automatically rotated into a new orientation, randomly sampled from all possible orientations, and a new target LED was switched on.

Motion tracking (Polhemus Liberty) was used to record movements of the right hand using three sensors attached to a participant’s right index finger, little finger, and wrist. The position and orientation of each sensor was sampled at 240 Hz and stored for off-line analyses.

### TMS procedures

The TMS procedures considered two experimental factors related to the location and timing of the TMS intervention. The factor tms-site had three conditions (EBA, IPS, SHAM). The experiment was set up to use active sites (EBA, IPS) in a factorial design, with a passive control (SHAM) for reference. The difference between EBA and IPS conditions captures site-specific effects of active stimulation, while the SHAM condition captures general (acoustic) effects of coil discharge. The factor tms-time had two conditions (EARLY, LATE). In relation to the factor tms-site, during the intake session, participants’ heads were coregistered to their individual structural magnetic resonance scans using TMS Navigator neuronavigation software (version 2.2.0, Localite). The skull locations with the minimal distance from the desired target locations (EBA, IPS) were estimated by the Localite software, and those two distances were used to define a subject- and site-specific stimulator output (see below in this subsection). The desired EBA location was determined according to a participant-specific fMRI localizer (see Functional localization of EBA). The average (SD) MNI coordinates of the EBA locations across the group were [−48 (2.4), −77 (4.0), 7 (4.8)]. EBA was stimulated in an inferior–superior direction. The desired IPS location was based on coordinates from a previous study (Zimmermann et al., 2012) that involved this portion of the posterior parietal cortex in state estimation during action planning (MNI coordinates, −22, −60, 58). IPS was stimulated in an inferior/posterior–superior/anterior direction, approximately perpendicular to the orientation of the intraparietal sulcus. MNI coordinates of the IPS target were transformed to subjects’ individual brain space using the inverse normalization parameters obtained during preprocessing of the MRI data. Coil orientation was slightly adjusted to optimize participants’ comfort, when necessary. SHAM TMS was implemented by using a coil tilted by 90° and interposed between the two coils targeting EBA and IPS (Fig. 1C). This coil configuration delivered an ineffective cortical stimulation, while closely matching the auditory stimulation produced by the other TMS coils. Participants wore ear plugs across the duration of the experiment.

The three coils were firmly held in place with mechanical arms (Manfrotto), and their location relative to the EBA and IPS targets was continuously monitored with the Localite software. At the beginning of each trial, if the head had moved >5 mm away from the desired configuration, the trial was delayed and participants were asked to move their head back to its previous position. Throughout the experiment, only two coils were simultaneously connected at any given time to the two available stimulators (model X100 stimulators, MagVenture). After each block of 50 trials, one of the previously active coils was disconnected, and the previously disconnected coil was connected. The order of active coils was counterbalanced over sessions and randomized over participants.

EBA and IPS stimulation was achieved with MagVenture MC-B65-HO Butterfly Coils with an average winding diameter of 55 mm, with a biphasic pulse shape. A C-B60 Butterfly Coil was used for SHAM stimulation. The stimulation intensity was customized for each site and participant, according to the following formula:

$$Intensity[\%]=50\%\;+\;2.4\%/mm\;\;*\left(dis\tan ce_{coil-t\arg et}-20\;\mathrm{mm}\right)$$

This formula was obtained through a pilot study performed in 10 independent participants using the same equipment. The pilot study, following the procedures of Stokes et al. (2005), indicated that the realized intensity at the target location can be kept stable by increasing stimulator output by 2.4% of maximum stimulator output (MSO) for each millimeter of additional space between the coil and the target location. Accordingly, we set the stimulator output to achieve 50% MSO at 2 cm from the coil, for each site and for each participant. The average (SD) stimulation intensity for EBA was 54% (7.0%) MSO, and for IPS it was 69% (7.3%) MSO. Stimulation intensity for the coil used for SHAM TMS was set to 100% MSO to closely match the sound level of the other TMS coils.

In relation to the factor tms-time, during the experiment, single TMS pulses were delivered at any time within two epochs of the interval between trial onset and release of the start button. A pilot study indicated that, on average, participants take about 600 ms to initiate their movement under the present experimental conditions. Accordingly, single-pulse TMS was delivered either EARLY during planning (100–300 ms after trial onset) or LATE during planning (300–500 ms after trial onset). No pulses were delivered if participants had already released the start button before the randomly selected time of stimulation had elapsed, and those trials were excluded from subsequent analyses.

### Measures of task performance

The effects of TMS on task performance were indexed with three parameters that captured features of motor preparation (planning time), of motor performance toward the initial bar configuration (grip choice), and of motor performance toward the desired bar configuration (goal-state error). All analyses were performed in MatLab (version R2009b, MathWorks) using *n*-way ANOVAs (function, anovan) with participant as a random factor. In the following section, we define the procedures used to quantify planning time, grip choice, and goal-state error.

Planning time was defined as the time between trial onset (when the bar and its desired orientation became visible) and movement onset. Movement onset was based on the motion-tracking data and was defined as the last local minimum in wrist velocity before peak velocity of the hand transport phase toward the bar (Schot et al., 2010).

Grip choice was defined as the probability of choosing a toward grip (i.e., that the bar was grasped with the thumb on the white end/toward the target LED) as a function of the initial orientation of the bar. Given human biomechanical constraints and end-state comfort effects, grip choice varies as a function of the initial and desired bar orientations, according to a logistic function (Rosenbaum et al., 2006). Accordingly, assessing the effects of TMS on grip choice requires a robust estimate of changes in the probability distribution of grip choices as a function of bar orientation. Estimating those changes involved three steps.

First, a grip probability function over the range of bar orientations (0–360°) was estimated by averaging single-trial grip choices over neighboring orientations with a moving Hanning weighting window (width, 45°; Fig. 2A). Second, we created the condition for pooling data across participants by phase adjusting the grip probability functions across participants, such that grip probability was consistently high at 0° and low at 180°. This was done by fitting a cosine with one free parameter, the phase, to the moving average, and then shifting the data by the phase parameter of the cosine (Fig. 2A). Third, the data were fitted to a psychometric function designed to parameterize grip choice biases (Fig. 2B) according to a least-squares approach. The psychometric function was as follows:

$$GRIP\;CHOICE(\varphi )=\alpha \;*\;\left(f_{fall}(\varphi )+f_{rise}\left(\varphi \right)\right)+\beta$$

with the following:

$$f_{fall}\left(\varphi \right)=1-\frac{1}{e^{\left(-\gamma_{fall}\;*\;\left[\phi \;–\;\delta_{fall}\right]\right)}}$$

$$f_{rise}\left(\varphi \right)=\frac{1}{e^{\left(-\gamma_{rise}\;*\;\left[\phi \;–\;\delta_{rise}\right]\right)}}$$

Grip choice is a function of orientation (φ; Fig. 2B), which was estimated as the sum of two logistic curves, one falling (*f*_fall_) and the other rising (*f*_rise_), with the following six free parameters: amplitude (∝) and offset (β), plus separate slope (γ_i,_ γ_2_) and phase-shift (δ_1,_ δ_2_) parameters for each logistic curve. These parameters provide robust and interpretable estimates of factors that influence grip choice as a function of bar orientation.

The amplitude parameter (∝; range, [0, 1]) measures to what extent grip choice is influenced by the expected body posture toward the end of the action (in short, ∝ = posture bias). A large ∝ value indicates a strong end-state comfort effect; namely, a consistent preference in grasping the bar with one grip type within a range of goal orientations, and with the opposite grip type within a complementary range of goal orientations. A small ∝ value indicates a weak end-state comfort effect; namely, either a stereotyped or a random grip type irrespective of goal orientation. The offset parameter (β; range, [−0.5, 0.5]) accounts for biases toward either grip type irrespective of goal orientation (in short, β = systematic bias). A large β value (±0.5) indicates that a participant grasps the bar such that his thumb is always pointing toward the target LED at the end (or away, for β → −0.5). A small β value indicates a weak systematic bias in grip choice. The slope parameters (γ_fall_ and γ_rise_) measure the range of goal orientations over which participants switch their grip preference from one type to the opposite grip type (in short, 1/γ_i_ = switch range; range, [1, 180]). The slope phase offset parameters (δ_fall,_ δ_rise_; range, [0, 360]) indicate the orientations at which participants switch their grip preference from one type to the opposite grip type.

Goal-state error was defined as the absolute (unsigned) difference (in degrees) between the desired orientation of the bar (as indicated by the target LED) and the final orientation of the bar.

### Statistical analyses of behavioral outcomes

Statistical analyses were based on correct trials (i.e., error trials with technical errors, movement initiation before TMS pulse or slower than 1000 ms, and/or wrong or incomplete actions were removed). Outliers were also removed [cutoff, path length and/or movement time four interquartile ranges (Q3–Q2) above the median]. Statistical inferences are based on a two-tailed false-positive rate of *p* < 0.05. There were two main analyses. First, the effect of the experimental conditions on planning times and goal-state error of multiple-option rotation trials was tested with a 3 × 2 two-way ANOVA with factors tms-site [EBA, IPS, SHAM] and tms-time [EARLY, LATE]. The goal of this analysis was to test whether the timing and the location of the TMS intervention modified motor performance. In order to qualify the specificity of the finding in relation to movement selection demands and the complexity of end-posture anticipation, this analysis was subsequently expanded to include action-type [HOLD, ROTATE] and grip-options [SINGLE, MULTIPLE]. Second, the effects on grip choice (indexed by posture bias, systematic bias, and switch range) were tested with a 3 × 2 full-factorial ANOVA, with factors tms-site [EBA, IPS, SHAM] and tms-time [EARLY, LATE], and subsequently expanded to include action-type [HOLD, ROTATE]. The goal of this second analysis was to test whether the timing and the location of the TMS intervention modified the influence of the expected body posture at the end of rotation actions.

### Functional localization of EBA

Following an anatomical scan (T1-weighted MP-RAGE sequence: TR, 2300 ms; TE, 3.03 ms; voxel size; 1.0 × 1.0 × 1.0 mm; on a 1.5 T Avanto MR Scanner, Siemens), the EBA of each participant was localized with fMRI (using the same 1.5 T Avanto MR scanner, 32-channel head coil for signal reception, whole-brain T2*-weighted multiecho echoplanar images: TR, 2180 ms; TE(1) = 9.4 ms; TE(2) = 21.2 ms; TE(3) = 33.0 ms; TE(4) = 45.0 ms; voxel size, 3.5 × 3.5 × 3.0 mm; gap size, 0.5 mm). The EBA localizer used a set of previously validated stimuli. The stimuli included 20 pictures of human bodies without heads (http://pages.bangor.ac.uk/∼pss811/page7/page7.html), 20 pictures of human-made objects, as well as 20 phase-scrambled versions of those stimuli. Stimuli were presented in a blocked design (10 blocks per condition; 20 stimuli per block; stimulus presentation time, 300 ms; interstimulus time, 450 ms). Within each block, two identical stimuli were presented sequentially. Participants were instructed to detect these stimulus repetitions (one-back task) to ensure attention to the stimuli. Across trials, the location of the stimuli on the screen was randomly shifted (stimulus size, ∼10° visual angle, shifted by 3.5° horizontally/vertically). In addition to this EBA localizer (256 volumes over ∼10 min), we also acquired a resting-state fMRI scan (10 min) and a diffusion tensor imaging (DTI) scan (12 min). Resting-state and DTI scans are not part of this report.

The EBA localizer scans were analyzed using MatLab and SPM8 (Wellcome Trust Centre for Neuroimaging at UCL, London, UK). First, functional images were spatially realigned using a Whittaker–Shannon (sinc) interpolation algorithm that estimates rigid body transformations (translations, rotations) by minimizing head movements between the first echo of each image and the reference image (Friston et al., 1995a). Next, the four echoes were combined into a single volume. For this, the first 30 volumes of the time series of 256 volumes were used to estimate the best weighted echo combination in order to optimally capture the BOLD response over the brain (Poser et al., 2006). These weights were then applied to the entire time series. Subsequently, the time series for each voxel were temporally realigned to the acquisition of the first slice. Anatomical images were spatially coregistered to the mean of the functional images. Normalization parameters to transform anatomical images to a standard EPI template centered in MNI space (Ashburner and Friston, 1999) were estimated, but not applied. Instead, the inverse of the normalization matrix was used to transform the TMS target location for IPS from MNI coordinates into each individual subject space.

For each of the three image categories, square-wave functions were constructed with a duration corresponding to the block duration and convolved with a canonical hemodynamic response function and its temporal derivative (Friston et al., 1995b). Additionally, the statistical model included 18 separate regressors of no interest, modeling residual head movement-related effects by including the six rigid-body motion parameters (translations and rotations), as well as their first- and second-order temporal derivatives. Parameter estimates for all regressors were obtained by maximum-likelihood estimation, using a temporal high-pass filter (cutoff, 128 s), modeling temporal autocorrelation as an AR(1) process. Linear contrasts pertaining to the main effects of the design were calculated. EBA was identified by comparing activity during the “body” condition with activity during the “object” condition, providing locations for left and right EBA (Downing et al., 2001). The activation peak of left EBA was used as target location for TMS.

## Results

Subjects were instructed to grasp a bar that could be positioned in any orientation to achieve an instructed goal state (Fig. 1). On some trials, the subject had to rotate the object, on other trials just had to hold the bar (factor action-type: HOLD, ROTATE). The combination of orientation and rotation ensured that in some cases only a single-grip posture was comfortable, while other combinations allowed multiple courses of action to accomplish the trial (factor grip-options: SINGLE, MULTIPLE). TMS could be delivered over left EBA, a caudal section of left IPS, or as SHAM stimulation (factor tms-site: EBA, IPS, SHAM) at any time during action planning (factor tms-time: EARLY [100–300 ms after trial onset], LATE [300–500 ms after trial onset]). Subjects were able to execute the grasp-and-rotate task proficiently (6% errors, 4% outliers) with no differences in performance across the three sessions in terms of planning time (*F*_(2,39)_ = 0.62, *p* = 0.5) or goal-state error (*F*_(2,39)_ = 1.60, *p* = 0.2). The factor action-type (HOLD, ROTATE) influenced task performance, as indexed by planning time, goal-state error, and grip choice (Table 1). In the following section, we describe the effects of early and late TMS over EBA, IPS, and SHAM on participants’ performance as a function of action-type and grip-options.

**Table 1:** Fitted parameters (mean ± SD) and statistical differences (*t* values, *p* values) comparing the effects of HOLD and ROTATE conditions on planning time and goal-state error, as well as grip choice (according to the psychometric function described in the Materials and Methods subsection Measures of task performance)

Effects of action type on task performance	HOLD trials	ROTATE trials	HOLD vs ROTATE
Planning time (ms)	420 (20)	411 (18)	*t*_(21)_ = 2.471, *p* = 0.022
Goal-state error (°)	2.96 (1.14)	8.35 (3.26)	*t*_(21)_ = 7.250, *p* < 0.001
Grip choice			
Posture bias (∝)	0.967 (0.029)	0.671 (0.244)	*t*_(21)_ = 5.859, *p* < 0.001
Systematic bias (β)	0.021 (0.022)	0.235 (0.184)	*t*_(21)_ = 5.554, *p* < 0.001
Switch range (1/γ)	0.092 (0.014)	0.141 (0.076)	*t*_(21)_ = 2.876, *p* = 0.009
Phase difference (Δφ)	135.4 (71.8)	162.3 (18.5)	*t*_(21)_ = 1.929, *p* = 0.067

### Planning time

Planning times were influenced by tms-time and action-type, with longer planning when TMS pulses were delivered late during planning (early TMS, 392 ± 79 ms; late TMS, 440 ± 99 ms; *F*_(1,21)_ = 48.45, *p* < 0.001), and when participants held the bar (HOLD: 420 ± 20 ms; ROTATE: 411 ± 18 ms; *F*_(1,21)_ = 5.05, *p* = 0.036). Crucially, tms-site did not influence planning times, either as a main effect or in interaction with the other experimental factors (tms-time, action-type, or grip-options, all *p* > 0.10).

### Grip choice

The effects of TMS on grip choice were parameterized with three indexes (posture bias, systematic bias, and switch range) derived from a psychometric function fitted to the grip choices of each participant (see Measures of task performance). The posture bias quantifies to what extent grip choice is influenced by the expected body posture toward the end of the action. The systematic bias quantifies biases toward either grip type, irrespective of the bar orientation. The switch range quantifies the range of bar orientations over which participants switch their grip preference from one type to the opposite grip type. Please note that these parameters cannot be specified with respect to the factor grip-option as they consider the whole range of possible object orientations, spanning both single- and multiple-option configurations. The grip choice function accounted for a considerable amount of variance (mean *r* ^2^ = 0.918), and it was sensitive to variations in grip choice profiles induced by action-type (HOLD, ROTATE; see Table 1). For two subjects, the function could not be fitted for all conditions due to missing data for some orientations.

**Figure 3.**
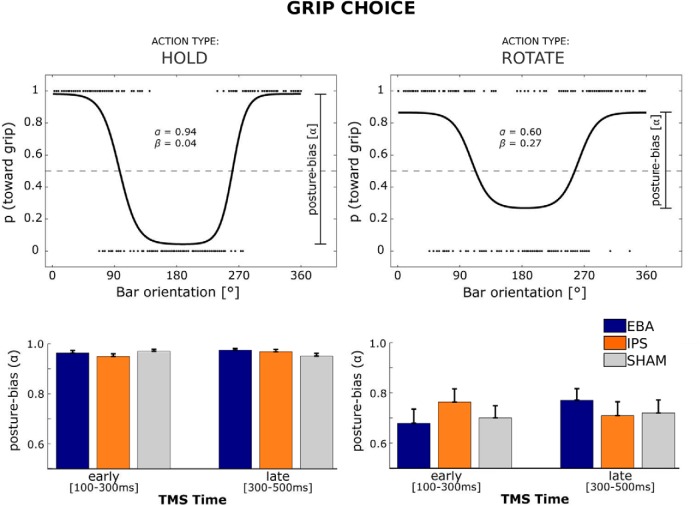
Effect of TMS on grip choice. Top, Example fits of grip choice of a single representative participant for action-type levels HOLD (left) and ROTATE (right), collapsed over levels of tms-site and tms-time. Dots represent single-trial grip choices (toward, away) after shifting by cosine phase (see Measures of task performance). Single-trial data are limited to 200 randomly selected trials per plot. Bottom, Posture bias (∝) parameters for action-type levels HOLD (left) and ROTATE (right) according to the psychometric function described in Measures of task performance. Error bars indicate standard error of the mean.

We adopted a factorial interaction design with two active TMS sites (EBA and IPS). For reference, we also included a SHAM stimulation condition to control for general effects. We were specifically interested in the effect of the timing and location of the TMS intervention on the influence of the expected body posture at the end of rotation trials (posture bias, ∝). Figure 3 illustrates that, in line with the findings on goal-state error, the effects of TMS intervention on EBA occurred when TMS was delivered early during the planning of rotation actions. Namely, when participants had to rotate the bar, the 3 × 2 interaction between tms-site [EBA, IPS, SHAM] and tms-time [EARLY, LATE] trended toward significance (*F*_(2,38)_ = 2.84, *p* = 0.071), driven by a significant difference in the effects evoked by TMS intervention over the active EBA and IPS sites (tms-site [EBA,IPS] × tms-time [EARLY,LATE]; *F*_(1,19)_ = 5.74, *p* = 0.027). Further exploration of this interaction indicated that, when applied early during planning, TMS over EBA led to a weaker posture bias, compared with TMS over IPS (EARLY TMS over EBA, ∝ = 0.68 ± 0.25; EARLY TMS over IPS, ∝ = 0.76 ± 0.24; *t*_(19)_ = 2.27, *p* = 0.035) or, as a trend, to LATE TMS over EBA (∝ = 0.77 ± 0.21; *t*_(19)_ = 1.92, *p* = 0.07). TMS over EBA and IPS could not be distinguished from TMS over SHAM (all *p* > 0.10). Moreover, the TMS effect was specific to ROTATE trials. Namely, the same effect was absent during HOLD trials (*F*_(1,19)_ = 0.29, *p* = 0.599), leading to a three-way interaction among tms-site, tms-time, and action-type on posture bias (*F*_(1,19)_ = 6.52, *p* = 0.02; Fig. 3). Finally, the specificity of this effect on posture bias was reinforced by the characteristics of the TMS effects on the other two parameters of grip choice. Namely, even though the three-way interactions among tms-site, tms-time , and action-type trended toward significance (systematic bias: *F*_(1,19)_ = 3.98, *p* = 0.06; switch range: *F*_(1,19)_ = 4.31, *p* = 0.052), those three-way interactions were not driven by differential effects of tms-site × tms-time interactions as a function of action-type.

### Goal-state error

Participants made larger errors during rotation actions [8.35 ± 3.26°; absolute difference between the desired (i.e., instructed) and the final orientation of the bar] than during holding actions (2.96 ± 1.14°; see Table 1). There were no main effects of tms-site or tms-time on goal-state error (all *p* > 0.10). Please note that, in contrast to the grip choice parameters, the goal-state error could be factorized according to SINGLE and MULTIPLE grip-options.

**Figure 4.**
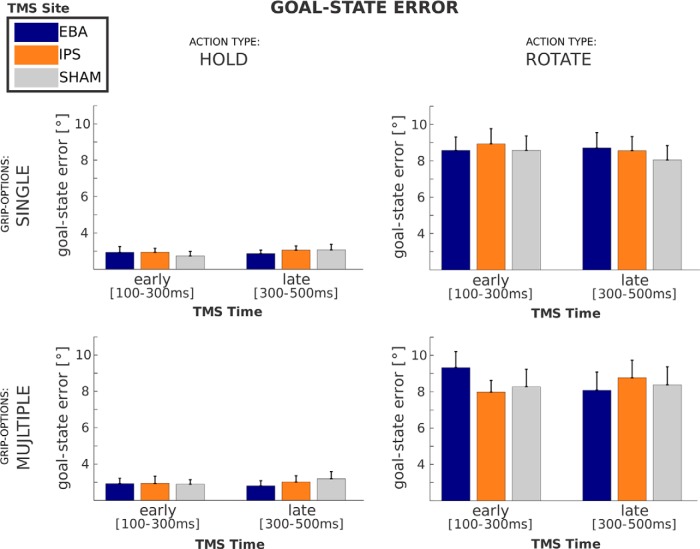
Effect of TMS on goal-state error. Absolute differences (in degrees) between the desired orientation of the bar (as indicated by the target LED) and the final orientation of the bar for HOLD actions (left column) and ROTATE actions (right column) with SINGLE (top row) and MULTIPLE (bottom row) grip-options. Error bars indicate standard error of the mean.

The main result of this study concerns the effect of timing and location of the TMS intervention on movement accuracy as a function of action-type and grip-options during multiple-option rotation trials. Figure 4 illustrates that the effect of TMS intervention was specific to the stimulation of EBA when TMS was delivered early during planning. Namely, the tms-site (EBA, IPS, SHAM) × tms-time (EARLY, LATE) interaction (*F*_(2,42)_ = 3.28, *p* = 0.047) was driven by larger goal-state errors following early stimulation of EBA compared with stimulation of IPS (*t*_(21)_ = 2.99, *p* = 0.007), as well as, as a trend toward significance, compared with SHAM TMS (*t*_(21)_ = 1.90, *p* = 0.07). Furthermore, the same interaction is not significant for single-option ROTATION trials, and single- and multiple-option HOLD trials (all *p* > 0.20), which is reflected in a significant four-way interaction among tms-site (EBA, IPS), tms-time (EARLY, LATE), action-type (HOLD, ROTATE), and grip-options (SINGLE, MULTIPLE) on goal-state error (*F*_(1,21)_ = 6.10, *p* = 0.02; Fig. 4). For reference, the 3 × 2 × 2 × 2 interaction that also considered SHAM TMS revealed a trend toward significance (*F*_(2,42)_ = 2.90, *p* = 0.066).

**Figure 5.**
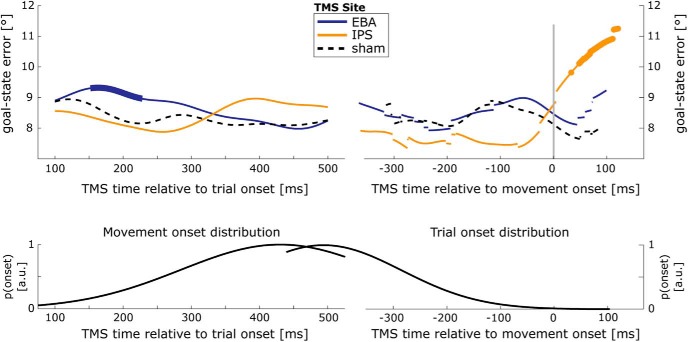
Time-resolved analysis of TMS effects on goal-state error. Top, Temporal dynamics of effects of TMS over EBA (blue) and IPS (orange) on goal-state error, time locked to trial onset (left) and wrist movement onset (right) for MULTIPLE-option ROTATE actions. Bold line sections indicate temporal clusters in which TMS over EBA/IPS had a significantly larger effect than sham stimulation at the same time. Abrupt transitions between datapoints of the time-resolved average are a consequence of the different number of participants contributing to different datapoints. Wrist movement onset refers to the earliest detectable sign of arm motion, namely, changes in wrist position (measured with a motion-tracking system) that occurred systematically earlier than the release of the home button. There were no TMS pulses delivered after participants released the home button. Bottom, Distribution of button press times and wrist movement-onset times (left) or trial-onset times (right) relative to TMS time in the corresponding top panel.

A *post hoc* exploration of the temporal dynamics of the effect of tms-time on goal-state error during multiple-option rotation trials assessed how this parameter changed as a function of the timing of the TMS intervention, relative to trial onset and movement onset. For every level of tms-site, a moving Hanning weighting window (width, 150 ms) was used to average goal-state errors over trials with similar relative TMS latencies. Subsequently, for every time point (bin size, 1 ms) these “time courses” for the stimulation of EBA and IPS were compared with the stimulation of SHAM using paired *t* tests with an α level of *p* < 0.05. Cluster-based permutation tests were performed in FieldTrip (Oostenveld et al., 2011) to correct for multiple comparisons, where applicable (i.e., not possible for movement-locked analyses due to changing numbers of participants contributing to different time points). When TMS intervention was time locked to trial onset (Fig. 5, left), the stimulation of EBA resulted in a significant cluster between 152 and 228 ms after trial onset compared with SHAM stimulation (*p* = 0.046). When time locked to wrist movement onset (Fig. 5, right), the stimulation of IPS from 48 ms after wrist movement onset onward (but before button release) resulted in significantly larger errors compared with SHAM stimulation.

## Discussion

This study considered the hypothesis that perceptual features represented in the ventral visual stream are used as a prior by the dorsal visuomotor stream to specify motor plans, especially when the action requires choices between different possibilities and the combination of multiple states. A prediction of this hypothesis is that transient alterations in ventral stream function should be particularly disruptive before the involvement of the dorsal visuomotor stream in motor planning of articulated actions chosen from multiple possibilities. This prediction was tested by comparing the effects of single-pulse TMS delivered to EBA, IPS, or SHAM, either early or late during the planning of grasping movements. Grip selection demands were manipulated by asking participants to grasp a bar and rotate it to orientations compatible with either a single- or multiple-grip configuration. Action- and posture-planning complexity were manipulated by asking participants to rotate the bar either marginally, to a goal close to its initial orientation, or majorly, to a nearly inverted goal. Under these circumstances, the choice of underhand or overhand grip is conditional on the biomechanical comfort of the end-state posture (Rosenbaum et al., 2001, 2012). Interfering with EBA activity had the following two main consequences: end-state posture had less influence on grip selection; and end-state accuracy decreased. These effects were temporally, spatially, and motorically specific. The effects were limited to interference delivered to EBA early during planning of actions requiring ∼180° rotations of the bar. Significantly weaker effects were evoked by the stimulation of EBA later during planning, early stimulation of IPS and SHAM, or stimulation during trials requiring marginal rotations of the bar. These observations qualify the content and function of the contribution of EBA to motor behavior, suggesting that EBA provides the dorsal visuomotor stream with a desired goal posture for actions that require anticipation of future postural states.

### EBA contribution to motor performance

Previous work has shown that the EBA is active during action preparation and motor imagery (Astafiev et al., 2004; Kühn et al., 2011; Zimmermann et al., 2012), but the necessity and content of EBA contributions to motor-related processes remain largely unknown and controversial (Urgesi et al., 2007; Downing and Peelen, 2011; Vangeneugden et al., 2014; Lingnau and Downing, 2015). This study adds three novel pieces of evidence to this debate. First, by using time-resolved interference, this study shows that EBA contributions to motor behavior are limited in time: they occur during the early phase of action planning (Figures 3, 4, 5). Second, by distinguishing the effects of postural comfort across different action epochs, this study shows that EBA contributions to motor behavior are about predictions of body postures, rather than the processing of current sensory material. Third, by manipulating the complexity of those predictions, this study shows that the contribution of EBA to motor behavior is particularly important when the predicted body posture needs to be assembled according to a task rule, rather than directly estimated from the available sensory evidence (Fig. 4).

These observations derive from effects on motor performance. However, the anatomical connectivity and response properties of EBA make it unlikely that those effects are driven by direct interference with the calculation of arm and hand movement vectors. It appears more plausible that disturbing EBA increases noise in the computation of the predicted body posture. This hypothetical effect would explain two features of the current data. First, increased noise in end-posture computation would correspondingly increase noise in the selection of a grip biomechanically appropriate for that desired posture (Rosenbaum et al., 2001). A more variable grip selection would become apparent as a reduced dependency of grip choice on end-posture configuration (posture bias; Fig. 3). Second, increased noise in the computation of the desired end posture would also result in a larger variation in the end state of the action (goal-state error; Figs. 4, 5).

These findings fit with the posture-based motion planning theory (Rosenbaum et al., 1995, 2001). The theory postulates that a posture appropriate for an action outcome is computed by combining posture primitives, and then that desired posture is used for calculating a forward model and a feedback control policy (Wolpert and Ghahramani, 2000; Shadmehr and Krakauer, 2008). The present findings suggest that EBA might implement those computations and provide the dorsal visuomotor stream with predictions on suitable postures for achieving the action outcome. Recent work (Bracci et al., 2012; Perini et al., 2014) has shown that an adjacent portion of the left lateral occipitotemporal cortex contains representations of body parts and tools, as well as associated actions. The present study suggests that these occipitotemporal representations are used during goal-directed actions.

### IPS contribution to motor performance

Previous work has shown that interference with posterior parietal regions late during planning or during action execution leads to decreased movement accuracy (Desmurget et al., 1999; Davare et al., 2012). The present findings fit with those observations. In this study, participants made larger movement errors when IPS was disturbed late during the planning of movements requiring 180° bar rotations and a choice between two equally favored grips (Fig. 4). Accordingly, it appears likely that TMS interfered with the computations of forward models and state estimation, known to be supported in this portion of the intraparietal sulcus (Wolpert et al., 1998; Shadmehr and Krakauer, 2008).

### Perceptuomotor interactions

The contribution of EBA to motor behavior is an instance of the long-standing issue of how perceptual processing interfaces with action planning (Faillenot et al., 1997; Milner and Goodale 2008). Some authors (Milner et al., 2001; Cohen et al., 2009) have argued that the neural integration of perceptual and visuospatial features is time consuming, and therefore only actions relying on extensive preparation can afford to achieve that integration. In fact, the evidence gathered in this study suggests that ventral stream areas might initialize motor planning with desired postural states. More generally, we speculate that perceptual information from ventral stream areas might provide priors for structuring the computations of forward models and feedback control policies computed in the dorsal visuomotor stream and in the cerebellum (Wolpert and Ghahramani 2000). It remains to be seen how these perceptuomotor interactions are anatomically and computationally implemented. For instance, it is unclear whether the postural configurations encoded in the occipitotemporal cortex (Bracci et al., 2012) are already in a format suitable for influencing sensorimotor computations implemented in the parietofrontal network. It is also unclear how the EBA is gaining access to that network. Recent work, also based on the planning of goal-directed movements, has highlighted the anterior portion of the intraparietal sulcus as a crucial hub for combining perception- and motor-related information (Verhagen et al., 2012). Empirical evidence on the connectivity of the human lateral occipitotemporal cortex will be crucial for qualifying those functional considerations.

### Interpretational issues

The task involved a large number of factors and outcome measures. However, despite the fact that the TMS intervention could have affected a number of planning and performance parameters, the TMS effects loaded on high-order variables of a psychometric model and were constrained by high-order interactions across experimental factors. For instance, the findings are bound to stimulation delivered during different epochs of movement planning. This feature of the results excludes general arousal effects associated with TMS (Duecker and Sack 2013). The findings pertain to trials requiring different rotations of the bar. This feature of the results excludes that TMS to EBA disrupted visual processing per se. However, some performance parameters were likely to be affected by non-neural side effects of TMS, most prominently the acoustic and somatosensory peripheral stimulation evoked by the delivery of the magnetic pulse. For instance, planning times increased with TMS pulse latency: movements were initiated later when TMS was applied later. This happened during both verum and sham stimulation, an indication that this effect, orthogonal to the main findings of this study, was driven by peripheral effects.

Whereas the effects of EBA relative to IPS provide reliable and significant results, the effects of sham stimulation, in interactions and specific contrasts, are often not clearly distinguishable from the stimulation of EBA and IPS. This suggests a (weak) opposite effect of IPS relative to EBA stimulation, which may have contributed to the statistical significance. Such an opposing effect fits with the overall interpretation of the roles of EBA and IPS. This study argues that EBA provides the motor system with a predicted goal posture according to high-level task-related preferences. It is already known that the lack of this end-state representation does not result in impairment in action execution. For instance, Dijkerman et al. (2009) showed that damage to the ventral stream areas (which might include EBA) does not prevent action planning, but results in atypical action selection. We argue that, without early input from EBA, dorsal stream areas (i.e., IPS) plan the grasping movement with a more random outcome (resulting in larger goal-state errors), given the lack of constraints on comfortable and effective action end states. At the same time, IPS interference at an early time in planning might reduce the ability to plan the grasping movement without those constraints imposed by EBA, and, in turn, facilitate the role of EBA. Such an effect of IPS interference would indeed contribute to the statistical significance of our results, but would not change their interpretation.

It might be argued that the spatial distribution of TMS effects is broad, and the effects of this study cannot be univocally associated with EBA. The experimental procedures suggest otherwise. EBA location was defined with fMRI on a subject-by-subject basis. The spatial accuracy of stimulation was continuously monitored and constrained to remain within 5 mm from the desired scalp location. Stimulation intensity was also customized, on a site- and subject-specific basis, to account for variations in the distance between coil and cortical target location. Moreover, the single-pulse intervention, at a relatively modest intensity, ensures that only neuronal populations directly under the coil that are already activated by the task are effectively perturbed, whereas neighboring or uninvolved neuronal populations are more likely to remain functionally unaffected (Allen et al., 2007). The different methods used to localize the TMS targets for EBA (subject-specific fMRI) and IPS (average stereotaxic coordinates from earlier studies) could, however, have introduced a bias toward stronger detection power for EBA stimulation compared with IPS stimulation (Sack et al., 2009).

The analysis involved some choices that may influence the results, for example, the separation of single- and multiple-option trials in a 75:25 ratio. However, these choices did not constrain the results. In fact, repeating the analyses across a range of single-option/multiple-option ratios led to similar, sometimes stronger, results for the interaction between tms-site and tms-time.

### Conclusions

This study shows that disrupting neuronal processing in EBA, early during action planning, causes alterations in goal-oriented motor behavior. We suggest that those observations can be interpreted in the context of a general mechanism in which ventral stream regions provide an initial structure to motor plans involving an articulate course of action chosen from multiple possibilities. Future studies will need to determine the sensory format and frame of reference of the representations used by the ventral stream to prime the sensorimotor transformations implemented in the dorsal stream.

## Synthesis

The decision was a result of the Reviewing Editor Trevor Drew and the peer reviewers coming together and discussing their recommendations until a consensus was reached. A fact-based synthesis statement explaining their decision and outlining what is needed to prepare a revision is listed below.

Reviewer #1

The manuscript is now much clearer and the design choices are better motivated. This is a carefully designed study with intriguing results.

Importantly, the sham data are now included in the analyses. These analyses show that many of the critical comparisons do not reach the 0.05 significance level relative to sham. This somewhat complicates the interpretation of the results: while the effects relative to IPS appear reliable, the effects relative to sham are much less clear. The pattern of results suggests that a (weak) opposite effect of IPS stimulation contributed to the statistical significance. The conclusions of the paper currently do not reflect this sufficiently, focusing almost exclusively on the causal role of EBA in title, abstract, and conclusions. At the very least, the Discussion should include a section discussing the sham results and how this speaks to the role of EBA vs the differential role of EBA vs IPS. Furthermore, sham conditions should be shown in figures 3 and 4 so that the reader can assess the degree to which EBA differed from sham in the various conditions.

Minor:

Worth mentioning in support of these findings: a recent paper showed that TMS over a region near EBA delayed responses to hand action/posture judgements associated with objects: Perini, Caramazza, Peelen. Front Hum Neurosci 2014.

Reviewer #2

This is an interesting paper for two reasons. First, the question is interesting -- whether occipitotemporal areas like EBA are involved in actions or only perception - and TMS provides a technique that enables stronger causal inference than techniques like fMRI. Second, it is an interesting case study to consider in terms of how to handle statistically marginal results. In sum, I really like the question; however, the answer is more uncertain that they manuscript portrays.

On the positive side, the question is interesting and timely and, if true, the results suggest a reconsideration of the role of EBA. In many ways, the experiment was designed nicely, with careful psychophysics, some appropriate controls (sham condition) and a reasonable number of participants given the (rather poor) norms in TMS. I am less concerned about some of the earlier comments than the reviewers (especially the need to analyze 90 deg trials as the design seems to make clear their purpose was avoid stereotyped responses not to be a key condition).

That said, there are some oddities in both the design and analysis:

1) It seems a strange methodological choice to employ a continuous range of time points for delivering TMS, which seems to unnecessarily add noise to a method that is pretty noisy to begin with. I kept wondering why the authors didn't just pick a small number of time points so as to have more statistical power at those points (and the manuscript could benefit from some added rationale here).

2) The other oddity is the insistence on emphasizing the differences between TMS effects at the two sites and downplaying of the sham control. This problem remains despite the fact that the issue was raised by both reviewers earlier. For example, the bar graphs still show only the comparisons between areas and don't plot data for the sham condition (Fig. 5 does but see point 4 below). No doubt this is because the comparisons with sham were only marginally significant. The authors need to be more forthright about the marginal significance of TMS to each area compared to the sham control (including plotting sham conditions in the bar graphs and a more explicit acknowledgement of this in the discussion).

3) Related to the above, despite the earlier comments of reviewers, the analysis still seems questionable. Most strikingly, there is a strange choice to analyze the data with a 3x2 ANOVA and then expand this to a 3x2x2x2 ANOVA (lines 300-318). Why not just go straight for the full four-factor ANOVA and be clear about the results.

4) The time analysis (Fig. 5) provides some value, but the use of p .05 for so many comparisons is questionable.

Minor points

The manuscript states, "TMS was delivered only when the TMS coils were positioned within 5 mm from the desired scalp locations." What is most relevant is not the scalp locations but the locations of the targeted cortical sites. Could these have moved more with respect to the coils (e.g., a slight change in angle could lead to the same skull site but different cortical targets)?

It should be acknowledged that differences in localization of the areas may lead to more statistical power to find effects in EBA than IPS. First EBA was targeted using subject-specific functional MRI localization; whereas IPS was targeted based on average stereotaxic coordinates from an earlier study in a different sample (see Sack et al., 2009, Journal of Cognitive Neuroscience). Second, in conjunction with the formula for adjusting TMS intensity to placement error, the statement (line 222) that "The average stimulation intensity (with standard deviations) for EBA was 54% (7.0) MSO, and for IPS it was 69% (7.3) MSO." indicates that the coil was typically further from the IPS site.
